# Low Testosterone and Semen Parameters in Male Partners of Infertile Couples Undergoing IVF with a Total Sperm Count Greater than 5 Million

**DOI:** 10.3390/jcm9123824

**Published:** 2020-11-26

**Authors:** Federica Di Guardo, Veerle Vloeberghs, Erlisa Bardhi, Christophe Blockeel, Greta Verheyen, Herman Tournaye, Panagiotis Drakopoulos

**Affiliations:** 1 Centre for Reproductive Medicine, Universitair Ziekenhuis Brussel, Vrije Universiteit Brussel, Laarbeeklaan, 101-1090 Brussels, Belgium; fediguardo@gmail.com (F.D.G.); veerle.vloeberghs@uzbrussel.be (V.V.); Erlisa.Bardhi@uzbrussel.be (E.B.); Christophe.Blockeel@uzbrussel.be (C.B.); Greta.Verheyen@uzbrussel.be (G.V.); herman.tournaye@uzbrussel.be (H.T.); 2 Department of General Surgery and Medical Surgical Specialties, Gynecology and Obstetrics Section, University of Catania, Via Santa Sofia 78, 95125 Catania, Italy; 3 Department of Obstetrics and Gynecology, Crete University, 70013 Crete, Greece

**Keywords:** testosterone, male infertility, IVF, semen analysis

## Abstract

Low serum testosterone is found in approximately 15% of subfertile men. Although testosterone is essential in spermatogenesis, it is unclear whether low testosterone levels may have a negative impact on the semen parameters of men belonging to infertile couples with a total sperm count greater than 5 million. Furthermore, it is debatable whether the initial evaluation of the subfertile male should include an endocrine assessment. This was a retrospective, single-center cohort study conducted at a tertiary fertility clinic. Male partners of infertile couples undergoing in vitro fertilization (IVF), with a total sperm count greater than 5 million, were included. All men provided morning blood samples, and none had been on exogenous testosterone or other relevant medications. Low total testosterone (TT) was defined as <264 ng/dL. Free T was calculated using TT and sex hormone-binding globulin (SHBG) levels (nmol/L) by a constant albumin concentration of 43 g/L. In total, 853 patients were included: 116 had low TT (<264 ng/dL) and 737 had normal TT (≥264 ng/dL). Semen volume, sperm cell count, progressive (A + B) motility and morphology (≥4% strict Kruger) were lower in the low TT group but not significantly different between low and normal TT groups (3.2 ± 1.79 vs. 3.23 ± 1.64, *p* = 0.87; 76.82 ± 83.18 vs. 67.55 ± 57.70, *p* = 0.7; 54.89 ± 19.45 vs. 56.25 ± 19.03, *p* = 0.6; 5.77 ± 3.23 vs. 6.89 ± 3.94, *p* = 0.23). The percentage of patients with below-reference sperm volume (<1.5 mL), cell count (<15 × 10^6^/mL), motility (A + B) (<32%) and morphology (<4%) was higher in the low TT group but not statistically different compared to the normal TT group. Multivariable regression analysis revealed that low TT and free T levels had no significant effect on the aforementioned semen parameters (coefficient: 3.94, 0.88, 1.37, 0.39; *p* = 0.53, 0.8, 0.3, 0.2; coefficient: 0.001, 0.06, 0.007, 0.0002; *p* = 0.73, 0.52, 0.85, 0.98). Despite our robust methodological approach, the presence of biases related to retrospective design cannot be excluded. Our findings highlighted the lack of association between low TT levels and semen parameter alterations in male partners of infertile couples undergoing IVF, with a total sperm count greater than 5 million. However, it is important to emphasize that more patients in the low TT group had subnormal semen parameters, albeit the difference was not statistically significant. Larger, prospective studies are warranted in order to validate these findings, as well as to investigate the existence of a TT threshold below which semen parameters might be negatively affected.

## 1. Introduction

Male factor infertility accounts for approximately 40–50% of all infertility cases [1], with studies reporting a significant overall decline in semen parameters over the last decade [2]. Although semen analysis remains the cornerstone of investigation of male factor infertility [3], its predictive value remains limited [4].

Low blood serum testosterone (T) has been observed in approximately 15% of infertile men [5]. T is the major androgen regulating spermatogenesis in the testis. It is produced by Leydig cells in response to the luteinizing hormone (LH) signal and acts as a paracrine factor by diffusion into the seminiferous tubules where the Sertoli cells are located [6]. Sertoli cells represent the primary target for T signalling, whose effects are mediated via the androgen receptor (AR) present in both nucleus and cytoplasm [7].

However, although evidence exists suggesting that T plays a crucial role in testicular function [8] and a minimum T level is required for spermatogenesis [9], it is questionable whether every man seeking medical help for infertility would actually benefit from an endocrine workup [5]. The American Society for Reproductive Medicine (ASRM) [10] and the European Association of Urology (EAU) [11] do not recommend endocrine testing as a primary first-line investigation and suggest that a total T (TT) test should only be performed under specific conditions, including low sperm concentration (particularly when the sperm concentration is <10 million/mL), sexual dysfunction or clinical findings of a specific endocrinopathy. However, others advocate that all infertile men merit an endocrine evaluation [5,12]. Recently, a post hoc analysis of a large randomized controlled trial (RCT) evaluating couples with unexplained infertility undergoing intrauterine insemination (IUI), revealed that low TT levels (<264 ng/dL) in the male partner were associated with poor sperm morphology [13].

In this scenario, the aim of our study was to investigate the effect of low TT blood serum levels on semen parameters in the male partner of couples with a sperm count greater than 5 million and undergoing in vitro fertilization (IVF) treatment.

## 2. Materials and Methods

### 2.1. Study Design

This was a retrospective, single-center cohort study including men with a total sperm count greater than 5 million among all consecutive infertile couples attending the Centre for Reproductive Medicine, Universitair Ziekenhuis Brussel, Belgium, from January 2009 to December 2017. Infertility was defined as the failure to achieve a clinical pregnancy after 12 months or more of regular unprotected sexual intercourse as reported by the World Health Organization (WHO) [14]. The study was approved by the Ethics Committee of Brussels University Hospital (approval B.U.N. 143201942575).

### 2.2. Study Population

The study population consisted of 853 male partners of all consecutive couples with primary infertility undergoing IVF and reporting a baseline semen analysis with a total sperm count greater than 5 million and at least 5 million motile sperm [13]. Men were recruited among couples that planned to undergo their first ovarian stimulation cycle for IVF, and each patient was only included once in the analysis. Our study included only asymptomatic men who provided an early morning blood sample (before 10 a.m.), as recommended by the Endocrine Society guidelines in order to avoid the natural diurnal variation in blood serum testosterone levels [15], and none had been on exogenous testosterone or other relevant medication such as aromatase inhibitors and/or antiestrogens. Male patients with the following characteristics were excluded from the study: history of secondary infertility; alcohol abuse; severe oligozoospermia; azoospermia; varicocele/hydrocele; postvasectomy reconstructive surgery; a history of genitourinary cancer; radio/chemotherapy; genitourinary tract active infections; genitourinary tract anomalies; injury to the testes or chronic diseases (such as diabetes, tuberculosis, hypertension, thyroid disease and chronic urinary tract infection). Patients were subdivided into two groups, depending on the blood serum TT levels: low (TT < 264 ng/dL) or normal (≥264 ng/dL), in line with the recently published guidelines of the Endocrine Society [15].

### 2.3. Semen Analysis

Semen collection took place after the blood sample. Sperm was collected in the laboratory by masturbation; 2–5 days of sexual abstinence were required. Only results from fresh ejaculates were used for the analysis, while frozen sperm samples were not considered. After 30 min to 1 h of liquefaction at 37 °C, semen analysis started with a macroscopic examination of the view, odor, viscosity, volume and pH. All semen samples were microscopically analyzed for sperm concentration, motility and morphology according to the criteria reported by the WHO in 2010 [16]. Sperm concentration was measured by a hemocytometer (improved Neubauer) on diluted samples. Only progressive motility (WHO grades A + B combined) was included, for more accuracy and consistency in results [17]. The Shorr staining procedure for sperm morphology was used.

### 2.4. Androgen Measurements

Serum sex hormone-binding globulin (SHBG) and TT were measured with the Elecsys Testosteron II and Elecsys SHBG assay on a Cobas 6000 immunoanalyzer (Roche Diagnostics, Mannheim, Germany). The same assay was used for all patients.

### 2.5. Statistical Analysis

Continuous data were presented as median (interquartile range)/mean ± standard deviation (SD) and categorical data were described in a number of ways, including the numerator and denominator, and percentages. Continuous variables were analyzed using the independent T-test or Mann–Whitney U test depending on the normality of the distribution. Normality was examined by the use of the Shapiro–Wilk test. Categorical variables were analyzed by Pearson’s chi-squared test or Fisher’s exact test, as appropriate. The association of TT status and free T with semen parameters (sperm volume, concentration, motility and morphology), after adjusting for potential confounders, was examined by multivariate linear regression. All covariates (male age, smoking status and SHBG) were simultaneously entered into the multivariate linear regression model. The assumptions for the final model were successfully tested. All statistical tests used a two-tailed α of 0.05. Analyses were performed using STATA 13.0. A *p*-value < 0.05 was considered as statistically significant.

## 3. Results

### 3.1. Patient Baseline Characteristics

In total, 853 male patients with a total sperm count greater than 5 million were included. Among them, 116 (13.6%) had low TT (<264 ng/dL) and 737 (86.4%) had normal TT (≥264 ng/dL). Patient baseline characteristics according to the blood serum TT levels are summarized in Table 1. LH, TT and SHBG were significantly lower in low TT patients compared to normal TT counterparts (3.43 ± 1.65 vs. 4.20 ± 2.04, *p* < 0.001; 213.37 ± 40.4 vs. 472.58 ± 141.02, *p* < 0.001, 33.8 ± 8.9 vs. 35 ± 14.7, *p* < 0.001, respectively). Conversely, age, blood serum follicle-stimulating hormone (FSH) and smoking status did not differ significantly between the two groups.

### 3.2. Semen Characteristics: Low TT vs. Normal TT

**Figure 1:**
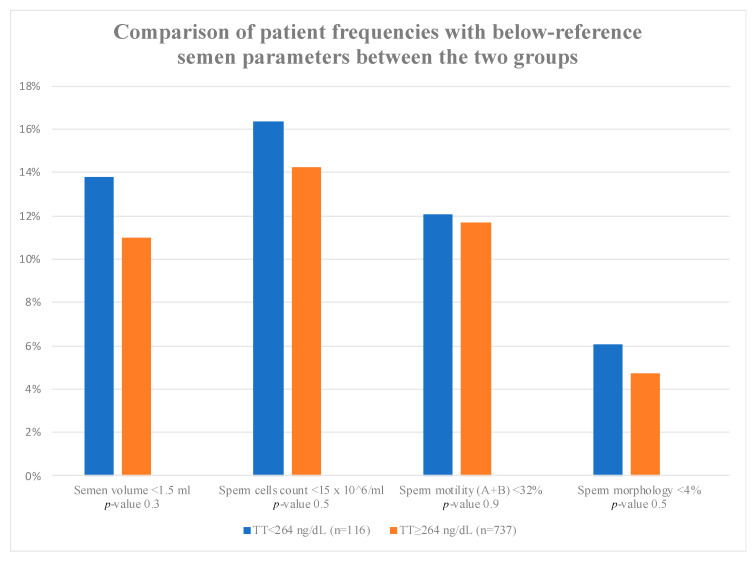
Values are expressed as percentages (%). The *p*-values were calculated using chi-squared test. *n* = number; TT = total testosterone. Semen volume (mL) > 1.5 mL = 16 (13.79%) in TT < 264 ng/dL vs. 81 (10.99%) in TT ≥ 264 ng/dL, *p*-value = 0.3. Sperm cell count (10^6^/mL) <15 × 10^6^ mL = 19 (16.38%) in TT < 264 ng/dL vs. 105 (14.25%) in TT ≥264 ng/dL, *p*-value = 0.5. Sperm motility (%) (A + B) < 32% = 14 (12.07%) in TT < 264 ng/dL vs. 86 (11.67%) in TT ≥264 ng/dL, *p*-value = 0.9. Sperm morphology (%) <4% = 7 (6.03%) in TT < 264 ng/dL vs. 35 (4.75%) in TT ≥264 ng/dL, *p*-value = 0.5.

Semen characteristics for the low TT and normal TT groups are displayed in Table 2. Total sperm number, semen sperm cell count and progressive motility (A + B) were comparable between the two groups (60.08 ± 18.94 vs. 61.95 ± 17.75, *p* = 0.34; 76.82 ± 83.18 vs. 67.55 ± 57.70, *p* = 0.7; 54.89 ± 19.45 vs. 56.25 ± 19.03, *p* = 0.6, respectively). Similarly, semen volume and morphology did not differ significantly (3.2 ± 1.79 vs. 3.23 ± 1.64, *p* = 0.87; 5.77 ± 3.23 vs. 6.89 ± 3.94, *p* = 0.23). The percentage of patients with below-reference volume (<1.5 mL), sperm cell count (<15 × 10^6^/mL), motility (A + B) <32% and/or morphology (<4%) was higher in the low TT group, although the difference when compared to the normal TT group was not significant (Figure 1). Moreover, the whole group was further divided into three subgroups (oligospermia, oligoasthenospermia and oligoasthenoteratospermia). The results did not show any significant difference between patients with normal and pathological TT levels (Supplementary Table S1).

### 3.3. Multivariable Regression Analysis

Multivariable regression analysis revealed that low TT did not have a significant effect on semen volume, sperm cell count, motility or morphology (coefficient: 3.94, 0.88, 1.37, 0.39; *p* = 0.53, 0.8, 0.3, 0.2, respectively) (Table 3). Similarly, free T levels were not significantly associated with semen volume, sperm cell count, motility or morphology (coefficient: 0.001, 0.06, 0.007, 0.0002; *p* = 0.73, 0.52, 0.85, 0.98, respectively) (Table 4). Results remained the same when testosterone was considered as a continuous variable.

## 4. Discussion

The results of our large retrospective study demonstrated that low TT (<264 ng/dL) was not associated with subnormal sperm parameters in infertile couples with a total sperm count greater than 5 million. Furthermore, despite the fact that the percentage of patients with below-reference sperm volume (<1.5 mL), sperm cell count (<15 × 10^6^/mL), motility (A + B) <32% and/or morphology (<4%) was higher in the low TT group than in the normal TT group, the difference was not statistically significant.

Few studies have investigated the impact of low TT on semen analysis so far. Our results were partly in line with a recent post hoc analysis of a large RCT evaluating couples with unexplained infertility undergoing IUI, which revealed that low TT levels (<264 ng/dL) in the male partner were not associated with low sperm parameters, except for poor sperm morphology [13]. However, the aforementioned study included men who performed blood draws in different moments of the day, without taking into account the diurnal secretion of T production in men [18,19,20]; therefore, men who had a blood test in the afternoon or evening could have been falsely identified as having low TT. Moreover, male partners may have been on treatment with drugs (i.e., selective estrogen receptor modulators or other androgenic agents) that could interfere with the blood serum TT concentrations. In the same vein, Uhler et al. (2003) evaluated 145 men belonging to reproductive age couples without known risk factors for infertility and observed no statistically significant correlation between blood serum T levels and semen parameters [21]. Nevertheless, this small study harbors a selection bias given that volunteers were only recruited via radio and newspaper advertising. Similarly, Hart et al. (2015) studied a large (365) group of young men with undefined fertility and did not find any correlation between TT concentrations and semen parameters. However, only men aged between 20 and 22 were included in the study [22]. Our results were in contrast with those reported by Keskin et al. (2015), who investigated the effect of serum gonadotropin and TT levels on the semen parameters of 382 patients that consulted a male infertility clinic [23]. The authors found a significant association between low TT levels and sperm motility and morphology, while no association was detected with semen volume and sperm concentration. However, the small sample size and methodological issues related to the study design made the authors advocate the conduction of larger-scale studies in order to solve the discrepancy between their findings and literature on the topic. Our study found that 13.6% of the infertile couples’ male partners with a total sperm count greater than 5 million had TT levels <264 ng/dL, in line with current evidence [15]. Blood serum TT is a surrogate marker for T concentration in the testes [23,24]. Indeed, intratesticular T levels were 30–100 times higher than blood serum levels [25,26,27,28,29], highlighting the necessity for higher T concentration in the testes in order for spermatogenesis to occur. Nonetheless, although a minimum T level for adequate human spermatogenesis is required [9], a threshold value has yet to be defined as it seems that even low blood serum TT levels may result in adequate intratesticular T concentrations for spermatogenesis to take place [23]. The role of intratesticular T is essential as it contributes to the initiation and maintaining of spermatogenesis, acting indirectly via Sertoli cells. T diffuses as a paracrine factor into the seminiferous tubules, where Sertoli cells are located, and supports the development of the final phases of spermatogenesis by controlling the secretion of proteins from these somatic testicular cells [30].

The strength of our study relies on its design, including a large sample size of infertile couples, with the male partner having a total sperm count greater than 5 million, performing the semen analysis in the same laboratory according to WHO 2010 guidelines. Moreover, the natural diurnal variation in blood serum testosterone levels was avoided due to the collection of patients’ blood samples during the early morning (before 10 a.m.). Despite the robust design of our study, limitations do exist and should be taken into consideration when interpreting the results. The retrospective nature of our study is associated with an inherent risk of bias. Therefore, although a significant effort has been made to eliminate all known sources of systematic error through multivariable analysis, nonapparent sources of bias might still exist. Pregnancy rates were not available for several couples. Moreover, data for the testosterone-to-estrogen ratio (T:E) were not available, although it has been reported to be an important parameter for the male fertility evaluation [31]. Finally, comparison of T levels with a male fertile group would allow a better understanding of the role of T in male reproduction.

The evaluation of other semen biomarkers such as the DNA fragmentation index or oxidative stress was not performed.

In conclusion, low male TT blood serum levels do not seem to have an impact on semen quality. However, it is important to emphasize that more patients in the low TT group had below-reference semen parameters. In the future, larger, prospective studies are warranted in order to validate these findings, as well as to investigate the existence of a TT threshold below which semen parameters are negatively affected. The next step would be to evaluate whether correcting T levels could have an impact on semen parameters and pregnancy rates and eventually examine the gene expression level of the sperm by using RNA sequencing.

## Figures and Tables

**Table 1:** Comparison of patient baseline characteristics between the two groups: low TT and normal TT.

	TT < 264 ng/dL (*n* = 116)	TT ≥ 264 ng/dL (*n* = 737)	*p*-Value
**Age**			
**Mean (SD)** **Median (IQR)**	34.42 ± 6.64 33.5 (30–37)	33.82 ± 5.39 33 (30–37)	0.9 ^a^
**Smoking status:**			
**Smokers** **Non smokers**	12 (10.34%) 104 (89.66%)	90 (12.21%) 647 (87.79%)	0.56 ^b^
**FSH** (IU/L)			
**Mean (SD)** **Median (IQR)**	4.04 ± 2.82 3.2 (2.6–4.3)	4.17 ± 2.91 3.5 (2.5–4.8)	0.4 ^a^
**LH** (IU/L)			
**Mean (SD)** **Median (IQR)**	3.43 ± 1.65 3.3 (2.1–4.7)	4.20 ± 2.04 3.8 (2.8–5.1)	<0.001 ^a^
**TT** (ng/dL)			
**Mean (SD)** **Median (IQR)**	213.37 ± 40.4 218.5 (192–243.5)	472.58 ± 141.02 450 (368–544)	<0.001 ^a^
**SHBG** (nmol/L)			
**Mean (SD)** **Median (IQR)**	33.8 ± 8.9 20.4 (17.8–29.3)	35 ± 14.7 32.3 (24.3–41.9)	<0.001 ^a^

Values are expressed as mean (SD) and median with interquartile range (IQR) (25–75%). ^a^ Mann–Whitney test. ^b^ Pearson’s chi-squared test. FSH: follicle-stimulating hormone (IU/L). LH: luteinizing hormone (IU/L). TT: total testosterone (ng/dL). SHBG: sex hormone-binding globulin (nmol/L).

**Table 2:** Comparison of the semen parameters between low TT and normal TT patients.

	TT < 264 ng/dL (*n* = 116)	TT ≥ 264 ng/dL (*n* = 737)	*p*-Value
**Semen volume (mL)**			
**Mean (SD)** **Median (IQR)**	3.2 ± 1.79 3.1 (2–4)	3.23 ± 1.64 3 (2–4.1)	0.87 ^a^
**Sperm cell count (10^6^/mL)**			
**Mean (SD)** **Median (IQR)**	76.82 ± 83.18 52.2 (27.5–110.75)	67.55 ± 57.70 54.3 (26.8–92.5)	0.7 ^a^
**Total sperm number (10^6^/ejaculate)**			
**Mean (SD)** **Median (IQR)**	60.08 ± 18.94 65 (53–75)	61.95 ± 17.75 64 (47–71)	0.34 ^a^
**Sperm motility (A + B) (%)**			
**Mean (SD)** **Median (IQR)**	54.89 ± 19.45 60 (43–68)	56.25 ± 19.03 59 (46–70)	0.6 ^a^
**Semen morphology (%)**			
**Mean (SD)** **Median (IQR)**	5.77 ± 3.23 5.5 (3–8)	6.89 ± 3.94 6 (4–10)	0.23 ^a^

Values are expressed as mean (SD) and median with interquartile range (IQR) (25–75%). ^a^ Mann–Whitney test.

**Table 3:** Multivariable regression analysis for the effect of low TT on semen parameters after adjustment for age, smoking status and SHBG.

Low TT	Coefficient	SE	*p*-Value
**Sperm cell count**	3.94	6.37	0.5
**Motility (A + B)**	0.88	3.58	0.8
**Morphology**	1.37	1.32	0.3
**Semen Volume**	0.39	0.31	0.2

SE: Standard Error.

**Table 4:** Multivariable regression analysis for the effect of free testosterone on semen parameters after adjustment for age, smoking status and SHBG.

Free T	Coefficient	SE	*p*-Value
**Volume**	0.001	0.003	0.73
**Sperm cell count**	0.06	0.10	0.52
**Motility (A + B)**	0.007	0.03	0.85
**Morphology**	0.0002	0.01	0.98

SE: Standard Error.

## Supplementary Materials

The following data are available online at https://www.mdpi.com/2077-0383/9/12/3824/s1, The whole group was divided into three subgroups (oligospermia, oligoasthenospermia and oligoasthenoteratospermia). Table S1 shows comparison of patient frequencies with oligospermia, oligoasthenospermia and oligoasthenoteratospermia between the low TT and normal TT group. The results did not show any significant difference between patients with normal and pathological TT levels.
