# Supplementary material for: Low Testosterone and Semen Parameters in Male Partners of Infertile Couples Undergoing IVF with a Total Sperm Count Greater than 5 Million
Source: J Clin Med. 2020 Nov 26;9(12):3824. doi: 10.3390/jcm9123824 (PMC7761260; doi:10.3390/jcm9123824)
Supplement: Supplementary file 1 [file jcm-09-03824-s001.pdf]

Supplementary Table I. Comparison of patient frequencies with Oligospermia, Oligoasthenospermia, Oligoasthenoteratospermia between the two groups

	**TT<264 ng/dL (*n*=116)**	**TT≥264 ng/dL (*n*=737)**	**P-value**
**Oligospermia**	97 (83.62%)	632 (85.75%)	0.54 [b]
**Oligoasthenospermia**	9 (7.76%)	46 (6.24%)	0.53 [b]
**Oligoasthenoteratospermia**	0 (0%)	5 (0.68%)	>0.99 [c]

Values are expressed as Number (*n*) and Percentage (%).

P value were calculated using [b] Chi-Squared test and [c] Fisher exact test
